# Meta-Heart Team: Enhancing Multidisciplinary Heart Teams Through Virtual Reality in the Metaverse

**DOI:** 10.1016/j.jacadv.2024.101454

**Published:** 2024-12-14

**Authors:** Ioannis Skalidis, Panagiotis Antiochos, Anastasios Apostolos, Kostantinos Toutouzas, Grigorios Tsigkas, Anna Giulia Pavon, Konstantinos S. Mylonas, Yiannis S. Chatzizisis, Panos Vardas, Dimitri Arangalage

**Affiliations:** a School of Medicine, University of Crete, Heraklion, Greece; b Lausanne University Hospital, Lausanne, Switzerland; c National and Kapodistrian University of Athens, Hippokration General Hospital of Athens, Athens, Greece; d University Hospital of Patras, Patras, Greece; e Cardiocentro Ticino Institute, Ente Ospedaliero Cantonale, Lugano, Switzerland; f University of Rochester Medical, Rochester, New York, USA; g Miller School of Medicine, University of Miami, Miami, Florida, USA; h Hygeia Hospitals Group, HHG, Biomedical Research Foundation Academy of Athens (BRFAA), Athens, Greece; i Bichat-Claude Bernard Hospital, Paris, France; j Ecole de Medicine, Université Paris Cité, Paris, France

Cardiovascular multidisciplinary heart teams (MDHTs) play a critical role in managing valvular heart diseases, with their importance steadily growing.^1^ However, challenges such as limited subspecialty expertise and time constraints hinder their effectiveness. While tertiary hospitals are better equipped, many smaller institutions struggle to form optimal MDHTs, affecting patient care. At the same time, the concept of the metaverse is emerging in clinical medicine.^2^ This study aimed to assess the feasibility of conducting the first international Meta-Heart Team session using virtual reality (VR).

The Meta-Heart Team participants were selected to encompass various medical specialties and countries. They were provided access via Oculus Quest-2 headsets (Meta) and web browsers. Digital avatars were generated to enhance the immersive nature of the virtual experience and to offer participants a personalized way to interact. Two participants were tasked with presenting the example cases and guiding the discussions, while the remaining 8 actively participated in the collaborative discourse.

A three-dimensional, hospital-inspired environment was created on the Aimedis metaverse platform, specifically designed to facilitate real-time multidisciplinary collaboration. The Meta-Heart Team room was equipped with advanced display technology to support interactive data review and collaborative analysis. Four large, high-resolution screens were strategically positioned around a central virtual conference room, each screen configured to project patient data in high clarity, including coronary angiograms, echocardiograms, and computed tomography scans. These screens featured zoom-in capabilities for detailed viewing, enabling participants to closely inspect specific aspects of the data.

The room also featured customizable interface controls, allowing users to move freely within the space, select and highlight data points on screens, and view multiple data sets simultaneously. Real-time audio integration facilitated continuous verbal communication, while spatial audio provided a realistic experience by adjusting sound based on a participant's virtual location within the room. These technical components were designed to simulate the functional layout of a traditional MDHT room while leveraging VR capabilities to enhance interactivity and visual detail.

A 0 to 10 scale questionnaire assessed participant experiences in a VR meeting, covering technical quality, immersion, willingness to participate again, and overall experience. Data are presented as mean ± SD or number (%).

**Figure 1:**
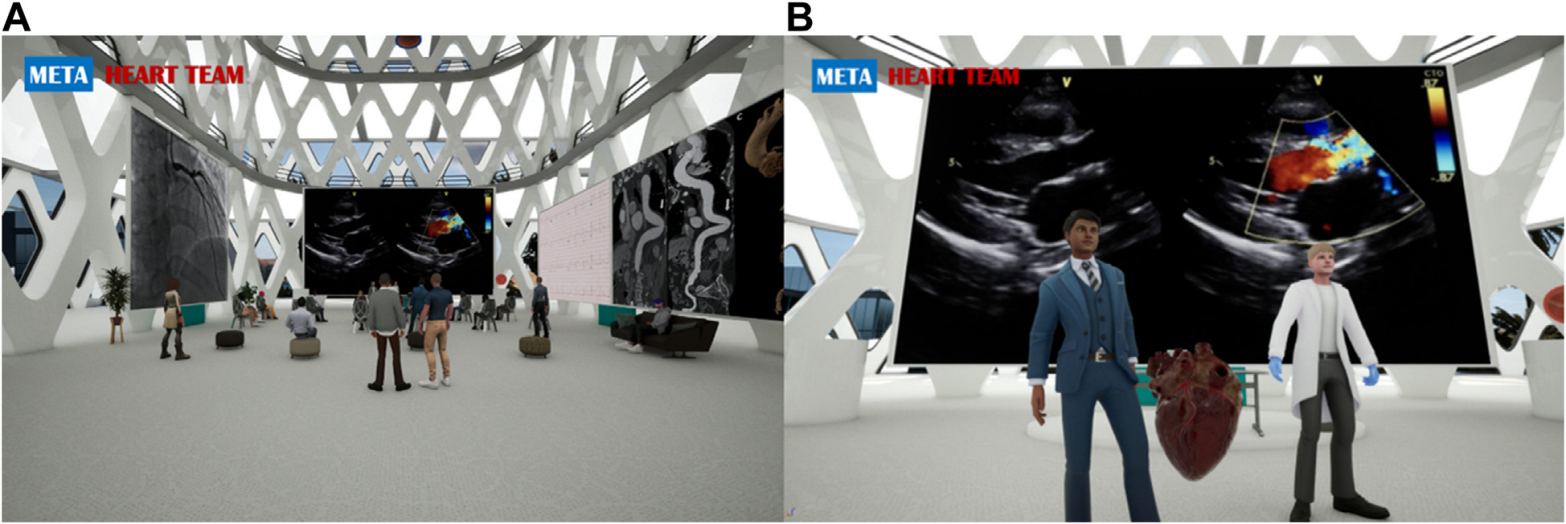
**Multidisciplinary Heart Teams in the Metaverse** (A) A virtual heart team room with four screens displaying key treatment information (coronary angiograms, echocardiography, electrocardiograms, and CT angiography) for participants. (B) Two presenters guide the meeting, offering detailed information and ensuring clear decision-making.

The virtual environment facilitated discussions among participants, enabling comprehensive analysis, including reviewing electrocardiograms, coronary angiograms, echocardiograms, and computed tomography scans. Three example valvular heart disease cases were discussed engaging 10 participants with 15 minutes dedicated to discussing each case. The multiscreen system allowed multidimensional and simultaneous examination of patient data (Figure 1.) The virtual environment demonstrated technical stability, with most participants reporting no technical impediments (90%). One participant (10%) experienced internet disconnection. Participants reported an overall positive experience (8.3 ± 1.6), emphasizing the technical quality (8.0 ± 1.8), good immersive experience (8.0 ± 1.3), willingness to participate again (9.2 ± 0.8), high confidence rate in treatment decisions (80%), and noted that the VR session saved time compared to traditional in-person meetings (80%). Three participants reported digital fatigue, characterized by reduced concentration and mental exhaustion from prolonged virtual engagement, while 80% highlighted data privacy concerns.

VR technology enabled a virtual metaverse for MDHT sessions, allowing comprehensive discussions. Feedback was positive, with high ratings for technical quality, engagement, and immersion. Despite minor issues, the platform enhanced patient data analysis and showed potential to improve MDHT collaboration and care strategies.

This virtual approach offers global access, overcoming geographical barriers, especially benefiting smaller hospitals by connecting them to specialized expertise. For smaller health systems, the Meta-Heart Team’s multiscreen system allows in-depth analyses, unlike traditional conferencing with limited shared screen options, thus improving decision-making with real-time access to multiple examination images and videos. Although this system’s potential benefits are substantial, the initial cost of VR hardware and metaverse platform subscriptions may pose challenges for smaller institutions. However, as VR technology becomes more widespread, these costs are likely to decrease, improving accessibility.^3^

Haptic feedback integration is being tested to enable tactile sensations, enhancing presence and potentially allowing remote clinical examinations. Advancements in facial recognition and motion tracking could lead to more realistic avatars, bridging the gap between physical and virtual interactions.

Although VR technology presents a promising platform for MDHTs, several limitations persist. The immersive nature of VR may lead to digital fatigue and reduced concentration, especially during extended sessions, as noted by some participants. Data privacy remains a critical concern due to the sensitive patient information shared within virtual environments. Participants worried about potential risks of data breaches on the VR platform, underscoring the need for advanced encryption, stringent professional guidelines, and comprehensive reporting mechanisms to ensure confidentiality.^4^ Additionally, the requirement for high-quality VR hardware and stable internet connectivity may restrict accessibility, particularly in regions with limited resources or infrastructure. Such limitations highlight the need for further research and development to address these challenges and establish protocols for safe, inclusive, and efficient VR-based MDHT sessions.

Leveraging VR technology within the metaverse for MDHT sessions is feasible, offering global accessibility and enhanced analysis. While challenges remain, the Meta-Heart Team complements rather than replaces in-person interactions, ushering in a new era of collaborative decision-making in line with modern technological advancements.
